# The Correlation of Clinicopathological Features With the Status of Surgical Margins in Renal Cell Cancer Patients Following Nephron-Sparing Surgery: A Systematic Review and Meta-Analysis

**DOI:** 10.3389/fonc.2019.00648

**Published:** 2019-07-18

**Authors:** Lijin Zhang, Bin Wu, Zhenlei Zha, Hu Zhao, Jun Yuan, Yejun Feng

**Affiliations:** Department of Urology, Affiliated Jiang-yin Hospital of the Southeast University Medical College, Jiangyin, China

**Keywords:** renal cell cancer, nephron-sparing surgery, clinicopathological, factors, positive surgical margins, meta-analysis

## Abstract

**Objectives:** The aim of this study was to evaluate the correlation of various clinicopathological variables with positive surgical margins (PSMs) in renal cell cancer (RCC) patients after nephron-sparing surgery (NSS).

**Methods:** A systematic search of PubMed, EMBASE, Web of Science, and China National Knowledge Infrastructure (CNKI) was performed to identify studies that compared PSMs with negative surgical margins (NSMs) and were published up to December 2018. Outcomes of interest included perioperative and postoperative variables, and the data were pooled by odds ratios (ORs)/standard mean differences (SMD) with 95% confidence intervals (CIs) to evaluate the strength of such associations. STATA 12.0 software was used for all statistical analyses.

**Results:** Based on the inclusion and exclusion criteria, 13 studies including 47,499 patients with RCC were analyzed. The results showed that higher Furhman grade (pooled OR = 1.25; 95% CI: 1.14–1.37; *P* < 0.001), higher pathological stage (pooled OR = 2.67; 95% CI: 2.05–3.50; *P* < 0.001), non-clear cell RCC (non-ccRCC) histology (pooled OR = 0.78; 95% CI: 0.72–0.84; *P* < 0.001), and non-white race (pooled OR = 0.90; 95% CI: 0.82–0.99; *P* = 0.026) were significantly associated with high risk of PSMs. However, age (pooled SMD = 0.09; 95% CI: −0.01–0.20; *P* = 0.078), gender (female vs. male) (pooled OR = 1.04; 95% CI: 0.96–1.12; *P* = 0.377), tumor laterality (left vs. right) (pooled OR = 1.09; 95% CI: 0.84–1.42; *P* = 0.501), tumor focality (unifocal vs. multifocal) (pooled OR = 0.67; 95% CI: 0.23–1.90; *P* = 0.445), tumor size (pooled SMD = 0.03; 95% CI: −0.10–0.15; *P* = 0.685), and surgical approach (open vs. non-open) (pooled OR = 0.94; 95% CI: 0.62–1.42; *P* = 0.763) had no relationship with PSMs. Sensitivity analysis showed that all models were stable, and no publication bias was observed in our study.

**Conclusions:** The present findings demonstrate that the presence of PSMs was associated with higher Furhman grade and higher pathological stage. Additionally, non-white patients with non-ccRCC histology had a high risk of PSMs after NSS. Further multicenter and long-term follow-up studies are required to verify these findings.

## Background

Renal cell cancer (RCC), which accounts for 2–3% of all adult malignancies, is the most common renal carcinoma (1). It was reported that there were over 14,000 RCC deaths in the USA in 2018 (2). With the widespread use of ultrasonography and CT scan, there has been a substantial increase in the diagnosis of early kidney cancer in recent years (3). The primary findings indicate that nephron-sparing surgery (NSS) may offer better renal function preservation and a lower risk of cardiovascular accident (4). Therefore, NSS has been recommended as the treatment of choice for tumor size <7 cm in the American Urological Association (AUA) guidelines (5).

Just like radical nephrectomy, NSS should always aim at complete tumor resection. However, positive surgical margins (PSMs) still exist after NSS, even in robot-assisted NSS. The incidence of PSMs ranges from 0 to 10% in recent reported studies (6–8), and the management of patients who have a PSM during NSS is still unclear. Although some studies have suggested that PSMs may be simply an accidental pathological manifestation (9, 10), most studies have shown that PSMs are associated with worse overall survival and increased risk of local recurrence (8, 11, 12).

In recent years, some studies have analyzed the potential risk factors for PSMs after NSS. Several factors have been proposed as predictors, such as older age, tumor size, location, pathological stage, and Fuhrman grade (13). There are, however, controversial results regarding these parameters for patients with PSMs. Currently, there is no commonly accepted prognostic nomogram for clinicians. With this background of conflicting results, we conducted a meta-analysis to identify predictive factors in patients with PSMs and provide more information for patient counseling.

## Materials and Methods

### Search Strategy

We searched electronic databases, including PubMed, EMBASE, Web of Science, and China National Knowledge Infrastructure (CNKI), for published studies that analyzed the relationship between PSMs and clinical parameters in RCC patients following NSS. The deadline for publication was up to December 2018. The following search term combinations were used: “RCC,” “renal cell carcinoma,” “NSS,” “partial nephrectomy (PN),” “simple enucleation (SE),”and “PSM.” The language of the literature covered was restricted to English or Chinese. In addition, the reference lists of the identified studies were also examined. This study was conducted according to the guidelines of the Preferred Reporting Items for Systematic Reviews and Meta-Analyses (PRISMA) (14). According to the grading standards of the Oxford Evidence-Based Medicine Center (15), the level of evidence in the current study was III.

### Eligibility Criteria

The inclusion criteria were as follows: (1) all patients with RCC were pathologically confirmed; (2) treatment was limited to surgery (NSS, PN, and SE); (3) the study investigated the association between PSMs and clinicopathological features; (4) the study used a case control design for PSMs and negative surgical margins (NSMs); (5) the study reported sufficient published data, including odds ratios (ORs)/standard mean differences (SMDs) and their 95% confidence intervals (CIs). The exclusion criteria were: (1) abstracts, case reports, letters, editorial comments, reviews, and meta-analyses; (2) a lack of sufficient data for further analysis; and (3) studies with duplicate data. If the data overlapped across several different articles, only the most recent or the largest body of research was reviewed.

### Data Extraction

The data were carefully reviewed and extracted from the eligible studies by two authors (ZLZ and HZ) according to the criteria of study selection. Any disagreement between the reviewers was resolved by consensus. The following characteristics were collected from eligible studies: (1) basic study information: first author's name, year of publication, country, and study design; (2) basic patients' characteristic: sample size, patient's age, surgical approach, and follow-up time; (3) pathological information: tumor diameter, tumor type, pathological grade, and stage.

### Statistical Analysis

Pooled ORs and SMDs with 95% CIs were used to evaluate the association of PSMs and clinicopathological characteristics. An observed OR > 1 with *P* < 0.05 implied more advanced clinicopathological characteristics for PSMs. A SMD of 1 with *P* < 0.05 indicates a relatively stronger improvement in the PSM group. The heterogeneity test of pooled ORs and SMDs was conducted using Cochran's *Q*-test and Higgins **I**-squared statistic. ***I***^**2**^ values >50% indicated heterogeneity among studies. When significant heterogeneity was observed among the studies (***I***^**2**^ > 50%), a random-effect model was used; otherwise, we adopted a fixed-effect model. Sensitivity analysis was conducted by sequentially omitting one study to determine the influence of individual data on the stability of the pooled results. Publication bias was assessed by funnel plot visual inspection and statistically evaluated by Egger's tests (*P* < 0.05 was considered a statistically significant publication bias). All calculations were performed using STATA 12.0 software (Stata Corporation, College Station, TX). Two-tailed *p* < 0.05 was considered statistically significant.

### Quality Assessment

According to the Newcastle–Ottawa quality assessment scale (NOS) (16), two researchers independently assessed the quality of each study. The NOS scores were split into three dimensions: object selection, inter-group comparability, and outcome measurement. Nine stars was defined as a full score; 8–9 stars was considered as being of high methodological quality; and 0–7 stars was considered as being of poor quality.

## Results

### Search Results

**Figure 1:**
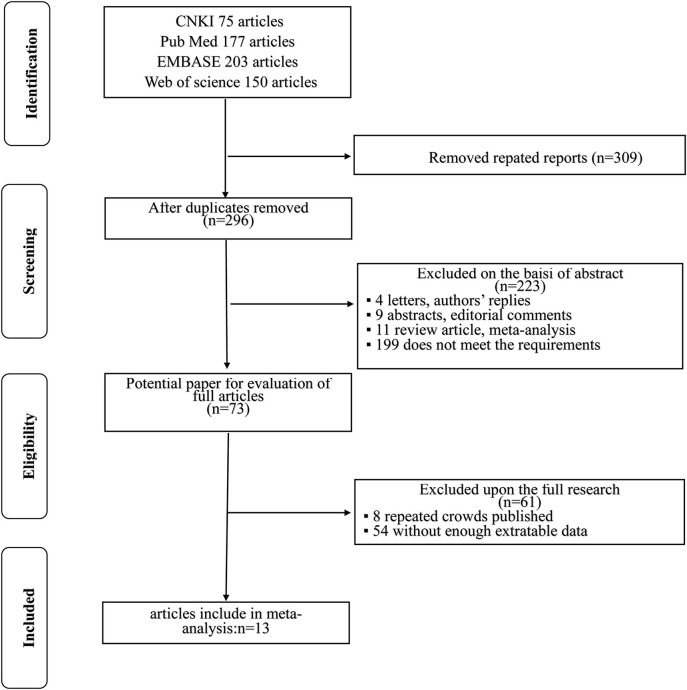
Flow diagram of the study selection process in this meta-analysis.

The flowchart for searching and screening the literature is shown in Figure 1. A total of 605 records were initially retrieved from the various electronic databases. A total of 309 duplicate reports were excluded. After screening the titles and abstracts, 223 articles were excluded for reasons such as letters, authors' replies, abstracts, editorial comments, reviews, and other obvious irrelevant studies. Subsequently, the 73 remaining full-text articles were assessed, and 13 were included in the final meta-analysis.

### Characteristics of the Included Studies

The included studies are all performed in recent years, and the main clinical and pathological characteristics of the included studies were listed in Table 1. The 13 included articles (17–29) were published from 2010 to 2018, and most of them came from North America (*n* = 7), Italy (*n* = 2), and Asia (*n* = 2). The mean sample size of patients per study was 3,654 (range: 134–20,762), and the follow-up of these studies ranged from 17.3 to 96 months. The patients in these studies were all diagnosed with RCC with different tumor types and received NSS treatment. All studies were written in English. Although the CNKI database was searched at the same time, no Chinese articles met the criteria for inclusion. The assessments of the NOS are presented in Table 2, and the results showed that all the studies were of high quality with scores ranging from 8 to 9.

**Table 1:** The main clinicopathological characteristics of all studies included in this meta-analysis.

**References**	**Country**	**Recruitment period**	**No. of patients**		**Age (years)**		**Stage 1–2/3–4**		**Grade 1–2/3–4**		**Tumor size (cm)**		**Follow-up (months)**
			**PSMs**	**NSMs**	**PSMs**	**NSMs**	**PSMs**	**NSMs**	**PSMs**	**NSMs**	**PSMs**	**NSMs**	
Tellini et al. (17)	Italy	1983–2014	27	432	Mean ± SD	Mean ± SD	25/2	416/16	NA	NA	Mean ± SD	Mean ± SD	Median (IQR)
					62.2 ± 10.2	60.7 ± 12.7					3.2 ± 1.3	3.1 ± 1.3	96 (74–131)
Shum et al. (18)	Indian	2004–2009	1,278	19,484	NA	NA	1,278/0	19,484/0	857/283	13,638/3,604	NA	NA	Median
													70.3
Petros et al. (19)	USA	1990–2015	34	100	Median (range)	Median (range)	27/7	92/8	22/11	64/26	Median (range)	Median (range)	Median
					59 (32–78)	62 (26–85)					3.1 (1.5–6)	3.0 (0.7–6.2)	62
Marchinena et al. (20)	Argentina	2010–2015	22	292	Mean ± SD	Mean ± SD	22/0	302/0	21/1	286/6	Median (IQR)	Median (IQR)	Median (IQR)
					58.9 ± 14.8	58.2 ± 12.2					2.7 (2.0–3.0)	3.0 (2.1–3.8)	24 (12–40)
Chen et al. (21)	USA	2010–2013	1,045	11,470	Mean	Mean	1,045/0	11,470/0	592/200	6,952/1,974	NA	NA	NA
					59	59							
Bansal et al. (22)	Canada	2011–2014	71	972	Median (IQR)	Median (IQR)	54/12	917/55	48/24	721/251	Median (range)	Median (range)	Median (IQR)
					61 (53–70)	61 (52–68)					3.0 (2.4–4.2)	3.0 (2.0–4.0)	19 (5–42)
Shah et al. (23)	USA	2006–2013	97	1,148	Mean ± SD	Mean ± SD	88/9	1,089/54	72/25	855/288	Mean ± SD	Mean ± SD	Median (IQR)
					59.7 ± 11.5	59.0 ± 11.9					3.3 ± 1.8	3.2 ± 1.6	33 (15–57)
Maurice et al. (24)	USA	2003–2006	302	5,736	Median (IQR)	Median (IQR)	285/17	5,613/123	258/44	4,855/851	Median (IQR)	Median (IQR)	Median (IQR)
					60 (50–69)	58 (48–67)					2.5 (2.0–3.5)	2.5 (1.9–3.5)	71 (56–85)
Kang et al. (25)	Korea	1999–2011	31	1,782	Mean ± SD	Mean ± SD	31/0	1,782/0	12/11	1,027/528	Mean ± SD	Mean ± SD	Median
					55.8 ± 11.4	53.8 ± 12.4					2.8 ± 1.1	2.5 ± 1.1	32.5
Schiavina et al. (26)	Italy	2009–2012	39	761	Mean ± SD	Mean ± SD	39/0	761/0	17/17	534/134	Median (IQR)	Median (IQR)	NA
					66.6 ± 8.8	61.8 ± 12.6					3.0 (2.2–4.0)	3.0 (2.4–4.0)	
Khalifeh et al. (27)	USA	2007–2012	21	922	Mean ± SD	Mean ± SD	20/1	878/44	11/10	546/376	Mean ± SD	Mean ± SD	Median (IQR)
					59.0 ± 11.9	59.0 ± 11.9					2.9 ± 1.1	2.9 ± 1.5	17.3 (6–41)
Ani et al. (28)	Canada	1995–2004	71	587	Mean ± SD	Mean ± SD	57/14	552/35	47/11	397/82	NA	NA	Median
					56.3 ± 14.8	57.7 ± 13.6							94.8
Bensalah et al. (29)	MC	1987–2006	111	664	Mean ± SD	Mean ± SD	97/14	619/45	75/32	535/129	Mean ± SD	Mean ± SD	Median
					61 ± 12.5	59.9 ± 12.6					3.5 ± 2	3.4 ± 1.8	37

*SD, standard deviation; IQR, inter quartile range; NA, data not applicable; PSMs, positive surgical margins; NSMs, negative surgical margins; MC, multi-centers*.

**Table 2:** Quality assessment based on the NOS of the included studies in this meta- analysis.

**References**	**Representativeness of the exposed cohort**	**Selection of the unexposed cohort**	**Ascertainment of exposure**	**Outcome of interest not present at start of study**	**Control for important factor or additional factor**	**Outcome assessment**	**Follow-up long enough for outcomes to occur**	**Adequacy of follow-up of cohort**	**Total quality scores**
Tellini et al. (17)	⋆	⋆	⋆	⋆	⋆	⋆	⋆	⋆	8
Shum et al. (18)	⋆	⋆	⋆	⋆	⋆⋆	⋆	⋆	⋆	9
Petros et al. (19)	⋆	⋆	⋆	⋆	⋆⋆	⋆	⋆	⋆	9
Marchinena et al. (20)	⋆	⋆	⋆	⋆	⋆	⋆	⋆	⋆	8
Chen et al. (21)	⋆	⋆	⋆	⋆	⋆⋆	⋆	⋆	—	8
Bansal et al. (22)	⋆	⋆	⋆	⋆	⋆	⋆	⋆	—	8
Shah et al. (23)	⋆	⋆	⋆	⋆	⋆	⋆	⋆	⋆	8
Maurice et al. (24)	⋆	⋆	⋆	⋆	⋆⋆	⋆	⋆	⋆	9
Kang et al. (25)	⋆	⋆	⋆	⋆	⋆	⋆	⋆	⋆	8
Schiavina et al. (26)	⋆	⋆	⋆	⋆	⋆⋆	⋆	⋆	—	8
Khalifeh et al. (27)	⋆	⋆	⋆	⋆	⋆⋆	⋆	⋆	—	8
Ani et al. (28)	⋆	⋆	⋆	⋆	⋆⋆	⋆	⋆	⋆	9
Bensalah et al. (29)	⋆	⋆	⋆	⋆	⋆⋆	⋆	⋆	⋆	9

*Nine stars was defined as a full score; 8–9 stars was considered as being of high methodological quality; and 0–7 stars was considered as being of poor quality*.

### Meta-Analysis

**Figure 2:**
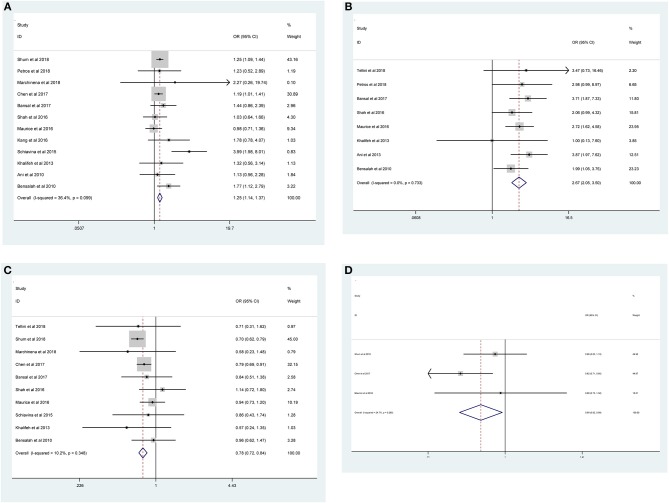
Forest plots of studies evaluating the prognostic factors for **(A)** higher Furhman grade, **(B)** higher pathological stage, **(C)** non-ccRCC histology, and **(D)** non-white race with PSMs.

**Figure 3:**
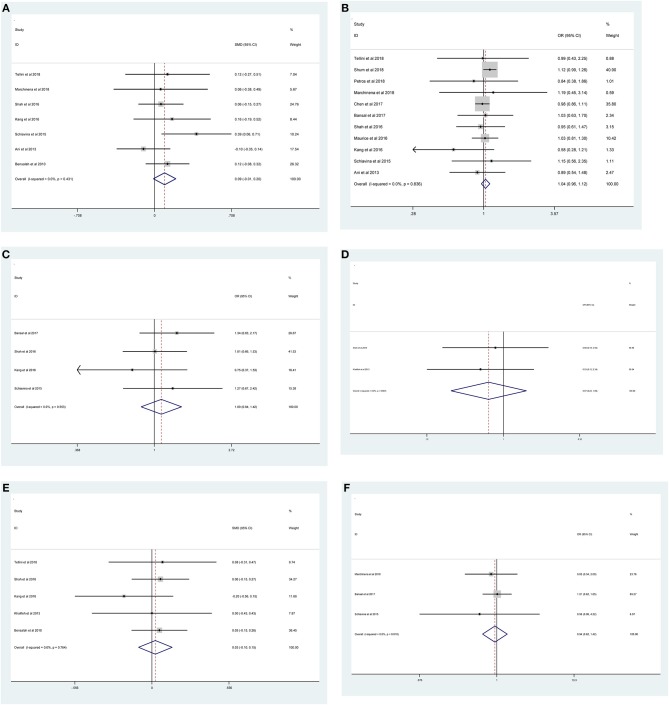
Forest plots of studies evaluating the association of PSMs and clinicopathological features in RCC patients. **(A)** age, **(B)** gender, **(C)** tumor laterality, **(D)** tumor focality, **(E)** tumor size, and **(F)** surgical approach.

As shown in Figure 2 and Table 3, the statistical result showed that PSMs were significantly correlated with higher Furhman grade (FE model, pooled OR = 1.25; 95% CI: 1.14–1.37; *P* < 0.001, Figure 2A), higher pathological stage (FE model, pooled OR = 2.67; 95% CI: 2.05–3.50; *P* < 0.001, Figure 2B), non-clear cell RCC (non-ccRCC) histology (FE model, pooled OR = 0.78; 95% CI: 0.72–0.84; *P* < 0.001, Figure 2C), and non-white race (FE model, pooled OR = 0.90; 95% CI: 0.82–0.99; *P* = 0.026, Figure 2D). However, age (FE model, pooled SMD = 0.09; 95% CI: −0.01–0.20; *P* = 0.078, Figure 3A), gender (female vs. male) (FE model, pooled OR = 1.04; 95% CI: 0.96–1.12; *P* = 0.377, Figure 3B), tumor laterality (left vs. right) (FE model, pooled OR = 1.09; 95% CI: 0.84–1.42; *P* = 0.501, Figure 3C), tumor focality (unifocal vs. multifocal) (FE model, pooled OR = 0.67; 95% CI: 0.23–1.90; *P* = 0.445, Figure 3D), tumor size (FE model, pooled SMD = 0.03; 95% CI: −0.10–0.15; *P* = 0.685, Figure 3E), and surgical approach (open vs. non-open) (FE model, pooled OR = 0.94; 95% CI: 0.62–1.42; *P* = 0.763, Figure 3F) were not significantly associated with PSMs. No great heterogeneity among studies was found, and therefore, the fixed-effect model was applied and no subgroup analysis was conducted in this meta-analysis.

**Table 3:** Summary of the meta-analysis for the associations between PSMs and clinicopathological features in RCC patients.

**Analysis specification**	**No. of studies**	**Study heterogeneity**		**Effects model**	**Pooled OR/SMD (95% CI)**	***P*-value**
		***I*^**2**^ (%)**	**P_**heterogeneity**_**			
**Age**						
Overall	7	0	0.431	Fixed	0.09 (−0.01, 0.20)	0.078
**Gender (female vs. male)**						
Overall	11	0	0.836	Fixed	1.04 (0.96, 1.12)	0.377
**Race (white vs. non-white)**						
Overall	3	24.7	0.265	Fixed	0.90 (0.82, 0.99)	0.026
**Laterality (left vs. right)**						
Overall	4	0	0.553	Fixed	1.09 (0.84, 1.42)	0.501
**Focality (unifocal vs. multifocal)**						
Overall	2	0	0.697	Fixed	0.67 (0.23, 1.90)	0.445
**Tumor size**						
Overall	5	0	0.764	Fixed	0.03 (−0.10, 0.15)	0.685
**Approach (open vs. non-open)**						
Overall	3	0	0.810	Fixed	0.94 (0.62, 1.42)	0.763
**Furhman grade (3–4 vs. 1–2)**						
Overall	12	36.4	0.099	Fixed	1.25 (1.14, 1.37)	<0.001
**Pathological stage (3–4 vs. 1–2)**						
Overall	8	0	0.733	Fixed	2.67 (2.05, 3.50)	<0.001
**Histology (ccRcc vs. non-ccRCC)**						
Overall	10	10.2	0.348	Fixed	0.78 (0.72, 0.84)	<0.001

### Sensitivity Analyses

Sensitivity analysis was conducted by removing individual studies in turn from our study. As shown in Supplementary Figures 1, 2, the pooled ORs for Furhman grade ranged from 1.17 (95% CI: 1.09–1.25) to 1.20 (95% CI: 1.11–1.30) (Supplementary Figure 1A), for pathological stage ranged from 2.33 (95% CI: 1.79–3.03) to 2.64 (95% CI: 2.03–3.44) (Supplementary Figure 1B), for gender ranged from 0.99 (95% CI: 0.95–1.03) to 1.02 (95% CI: 0.99–1.06) (Supplementary Figure 1C), for histology in non-ccRCC ranged from 0.92 (95% CI: 0.89–0.95) to 0.95 (95% CI: 0.91–0.98) (Supplementary Figure 1D), and for tumor laterality ranged from 1.00 (95% CI: 0.85–1.18) to 1.08 (95% CI: 0.94–1.24) (Supplementary Figure 1E). The pooled SMDs for age ranged from 0.06 (95% CI: −0.05–0.17) to 0.13 (95% CI: 0.02–0.25) (Supplementary Figure 2A), and for tumor size ranged from 0.01 (95% CI: −0.14–0.16) to 0.06 (95% CI: −0.07–0.18) (Supplementary Figure 2B). These results demonstrated that our meta-analysis was statistically stable. Due to the small number of the included studies, the sensitivity analysis for tumor focality, non-white race, and surgical approach was not valuable.

### Publication Bias

**Figure 4:**
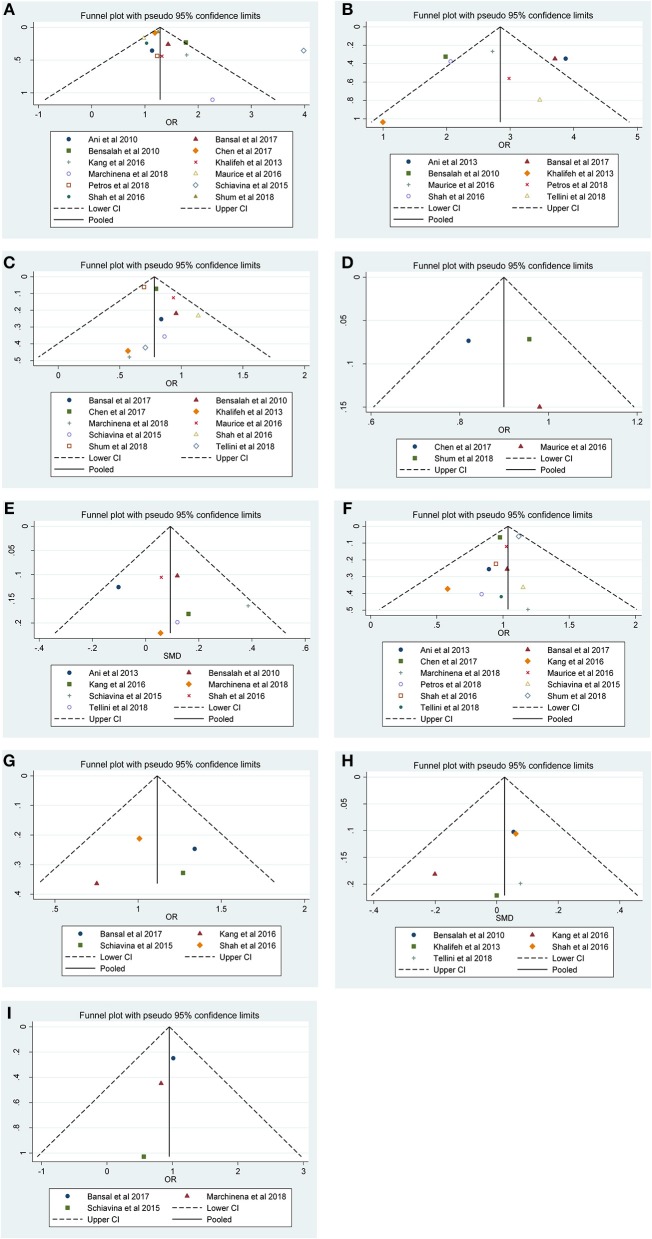
Funnel plots and Begg's tests for the evaluation of potential publication bias. **(A)** Furhman grade, **(B)** pathological stage, **(C)** non-ccRCC histology, **(D)** non-white race, **(E)** age, **(F)** gender, **(G)** tumor laterality, **(H)** tumor size, and **(I)** surgical approach.

Our results revealed that no evidence for significant publication bias was found for Furhman grade (*p*-Egger = 0.189, Figure 4A), pathological stage (*p*-Egger = 0.830, Figure 4B), non-ccRCC histology (*p*-Egger = 0.331, Figure 4C), non-white race (*p*-Egger = 0.765, Figure 4D), age (*p*-Egger = 0.527, Figure 4E), gender (*p*-Egger = 0.231, Figure 4F), tumor laterality (*p*-Egger = 0.811, Figure 4G), tumor size (*p*-Egger = 0.402, Figure 4H), and surgical approach (*p*-Egger = 0.154, Figure 4I). However, because the number of eligible studies in the current study was limited, the publication bias for tumor focality was not assessed.

## Discussion

Because of the recent advances in surgical techniques, NSS has been widely used as the “gold standard” for T1 RCC, and even for some complex tumors (30). According to the reported studies, there is a lower risk of renal failure associated with NSS, and it also provides a significantly higher quality of life to the RCC patient (4). Generally, there is a variety of options for NSS, including open NSS (30), hand-assisted laparoscopic NSS (31), and robot-assisted NSS (32). However, with the widely taken by NSS, a considerable part of positive surgical specimens with tumor cells in the final histopathology results were founded (11, 23). Therefore, the oncologic outcome in patients with PSMs represents a significant surgical and therapeutic challenge for the urologist.

PSMs have been recognized as an adverse prognostic sign for disease prognosis or local recurrence, and thus, NSS may theoretically decrease the probability of complete tumor resection (33). In previous research, the unfavorable effect of PSMs has been described for different types of cancers including: prostate (34), rectal (35), and bladder cancer (36). More recently, the clinical importance of PSMs in RCC patients after NSS was investigated. Khalifeh et al. (27) reported that PSMs on final pathological evaluation increased the hazard risk of recurrence and metastasis in 943 robot-assisted NSSs. Shum et al. (18) and Maurice et al. (24) identified PSMs as an independent risk factor for overall survival after PN based on the U.S. National Cancer Database.

The occurrence of PSMs in NSS may be associated with various clinicopathological factors. Because there are no randomized controlled experimental trials to generate sufficient clinical evidence, several factors have been suggested as predictors of PSMs. Schiavina et al. (26) found that older patients with higher Fuhrman grade had increased risks for PSMs. However, Ani et al. (28) used multivariable analysis to determine that age was not independently associated with PSMs (OR: 0.99, *p* = 0.3). Khalifeh et al. (27) also did not find any risk factors for PSMs related to tumor size, pathological stage, grade, or tumor focality. The reasons for the different outcomes described above may be related to differences in heterogeneity among studies. Therefore, further investigation for predicting prognostic factors for PSMs is still warranted. We aimed to identify predictors of risk stratification in patients with PSMs treated with NSS to better counsel patients.

Our study reports an overall incidence of PSMs of 6.6%, which is comparable to the other NSS results. To our knowledge, this is one of the largest meta-analyses evaluating the predictors of PSMs. Traditionally, the occurrence of PSMs after NSS was considered to be related to the nature of the RCC and the surgical procedure (37–39). In the current study, PSMs were statistically associated with Furhman grade (3–4 vs. 1–2), pathologic stage (3–4 vs. 1–2), non-ccRCC histology, and non-white race. The observations regarding Furhman grade and pathologic stage were in agreement with previously published studies (22). RCC includes several histologic types of tumors and each type of RCC has distinct clinical characteristics (40). Although ccRCC is the most common, the incidence of PSMs was significantly higher in patients with non-ccRCC. We also found that there may be a certain correlation between PSMs and ethnic differences. Similar to our results, Shum et al. (18) found that there were increased risks for PSMs in patients with papillary and chromophobe tumors compared to ccRCC. Chen et al. (21) reported that African-American patients who undergo robotic partial nephrectomy for localized RCC are at a higher risk for PSMs.

There are, however, some studies that reported that PSMs may be influenced by tumor size, fat invasion, tumor location, imperative indication, solitary kidney, and surgical technique (10, 27, 28, 33, 41, 42). In the present study, no correlations were founded among age, gender, tumor laterality, tumor focality, tumor size, or surgical approach. Compared with the above studies, we adopted a systematic analysis that contained as much relevant data as possible in order to make our results more persuasive. However, the biology of RCC in patients with PSMs may be quite different, and therefore, the dissemination of these results to all RCC patients should be carried out with discretion, as our findings might have some limitations.

Despite the comprehensive analysis of the factors for occurrence of PSMs, several limitations should be considered. First, like most studies of PSMs in RCC patients, the main limitation of our study is its retrospective nature. Second, the criteria to determine the histology, tumor, node, and metastasis (TNM) classification and PSMs were inconsistent in different studies. Additionally, most of the studies covered a period of 10–20 years after surgery, which may have generated heterogeneity of the overall results. Fortunately, no significant heterogeneity was found among studies when calculating the ORs/SMDs. Third, the number of included studies was limited in the sensitivity and publication bias analyses. Thus, studies with large samples are necessary to investigate the prognostic factors for PSMs. Our last limitation was that we retrieved relevant articles only through electronic databases, and all studies were in the English language, which may lead to language and selective bias, although no publication bias was detected in the current stage.

## Conclusion

In this study, we found that Furhman grade (3–4 vs. 1–2), pathologic stage (3–4 vs. 1–2), non-ccRCC histology, and non-white race are all independent predictors of PSMs. However, no correlations between age, gender, tumor laterality, tumor focality, tumor size, surgical approach, or PSMs were detected. Knowledge of the risk factors for PSMs may help clinicians to assess the incidence of PSMs following NSS, which can be used to improve treatment outcome. Given the current limitations in our meta-analysis, more multicenter prospective and longer-term follow up studies are required to validate our findings.

## Data Availability

All data generated or analyzed during this study are included in this published article.

## Author Contributions

LZ: project development and manuscript writing. BW: data collection and manuscript editing. ZZ and HZ: data collection. JY and YF: data analysis and data management.

### Conflict of Interest Statement

The authors declare that the research was conducted in the absence of any commercial or financial relationships that could be construed as a potential conflict of interest.

## Supplementary Material

Figure S1 Sensitivity analysis (pooled ORs) of the association between PSMs and clinicopathological factors in RCC patients. (**S1a**) Furhman grade, (**S1b**) pathological stage, (**S1c**) gender, (**S1d**) non-ccRCC histology, (**S1e**) tumor laterality.

Figure S2 Sensitivity analysis (pooled SMDs) of the association between PSMs and clinicopathological factors in RCC patients. (**S2a**) Age and (**S2b**) tumor size.
