# Supplementary figures and images for: The Correlation of Clinicopathological Features With the Status of Surgical Margins in Renal Cell Cancer Patients Following Nephron-Sparing Surgery: A Systematic Review and Meta-Analysis
Source: Front Oncol. 2019 Jul 18;9:648. doi: 10.3389/fonc.2019.00648 (PMC6657739; doi:10.3389/fonc.2019.00648)

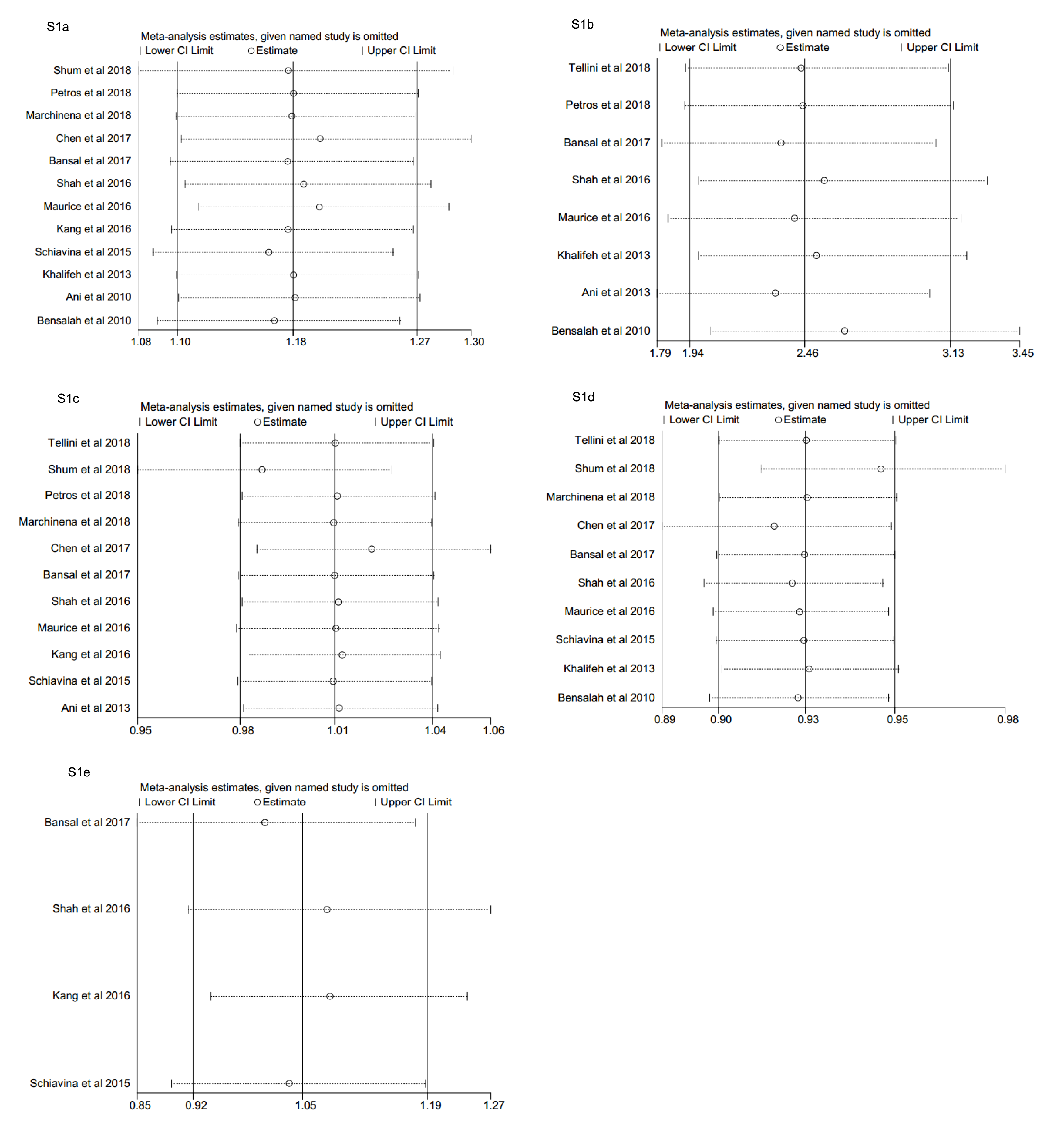

Supplement: Figure S1 — Sensitivity analysis (pooled ORs) of the association between PSMs and clinicopathological factors in RCC patients. (S1a) Furhman grade, (S1b) pathological stage, (S1c) gender, (S1d) non-ccRCC histology, (S1e) tumor laterality. [file Image_1.TIF]

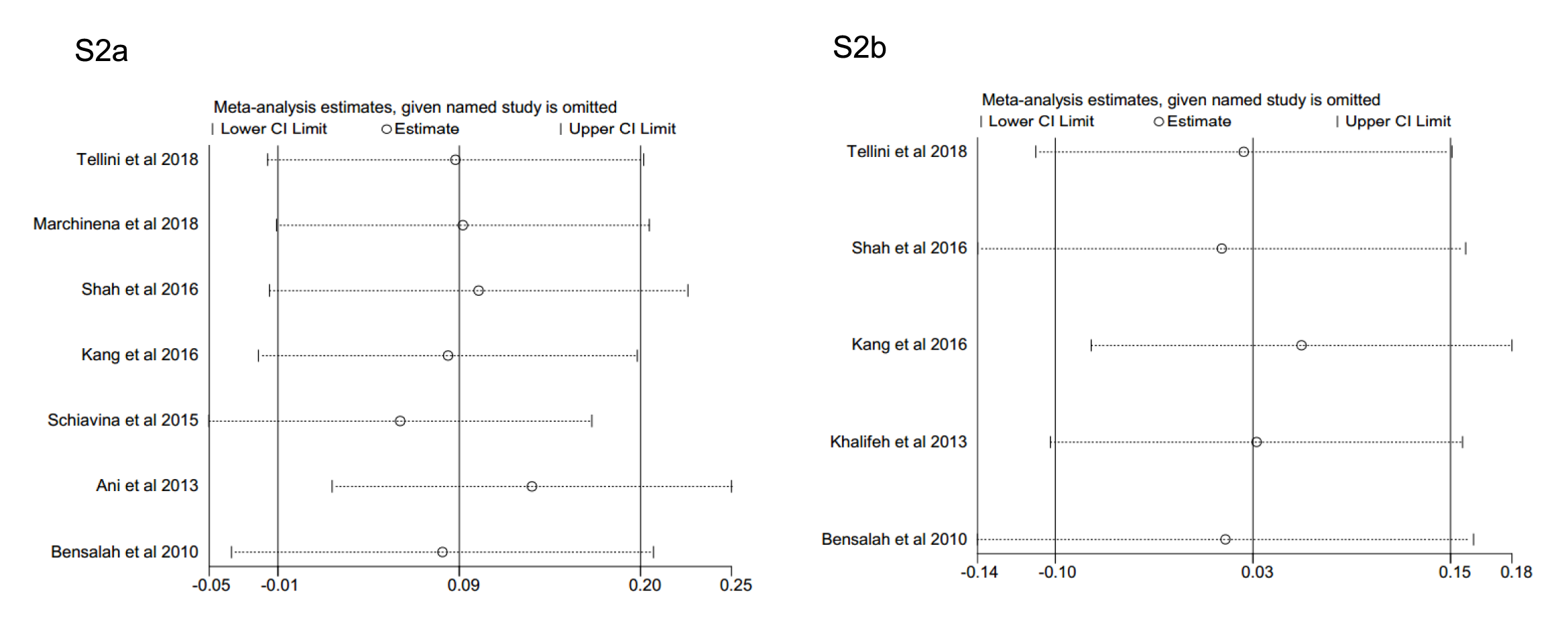

Supplement: Figure S2 — Sensitivity analysis (pooled SMDs) of the association between PSMs and clinicopathological factors in RCC patients. (S2a) Age and (S2b) tumor size. [file Image_2.TIF]
